# Mitochondrial Uncoupling Proteins (UCPs) as Key Modulators of ROS Homeostasis: A Crosstalk between Diabesity and Male Infertility?

**DOI:** 10.3390/antiox10111746

**Published:** 2021-10-30

**Authors:** Bruno S. Monteiro, Laís Freire-Brito, David F. Carrageta, Pedro F. Oliveira, Marco G. Alves

**Affiliations:** 1 Department of Anatomy and Unit for Multidisciplinary Research in Biomedicine (UMIB), Institute of Biomedical Sciences Abel Salazar (ICBAS), University of Porto, 4050-313 Porto, Portugal; b.monteiro@ua.pt (B.S.M.); laisbrito0330@gmail.com (L.F.-B.); davidcarrageta@gmail.com (D.F.C.); 2 Química Orgânica, Produtos Naturais e Agroalimentares (QOPNA) & Laboratório Associado para a Química Verde—Rede de Química e Tecnologia (LAQV-REQUIMTE), Department of Chemistry, University of Aveiro, 3810-193 Aveiro, Portugal; pfobox@gmail.com

**Keywords:** diabetes mellitus, male infertility, mitochondrial bioenergetics, obesity, oxidative stress, ROS production, UCPs

## Abstract

Uncoupling proteins (UCPs) are transmembrane proteins members of the mitochondrial anion transporter family present in the mitochondrial inner membrane. Currently, six homologs have been identified (UCP1-6) in mammals, with ubiquitous tissue distribution and multiple physiological functions. UCPs are regulators of key events for cellular bioenergetic metabolism, such as membrane potential, metabolic efficiency, and energy dissipation also functioning as pivotal modulators of ROS production and general cellular redox state. UCPs can act as proton channels, leading to proton re-entry the mitochondrial matrix from the intermembrane space and thus collapsing the proton gradient and decreasing the membrane potential. Each homolog exhibits its specific functions, from thermogenesis to regulation of ROS production. The expression and function of UCPs are intimately linked to diabesity, with their dysregulation/dysfunction not only associated to diabesity onset, but also by exacerbating oxidative stress-related damage. Male infertility is one of the most overlooked diabesity-related comorbidities, where high oxidative stress takes a major role. In this review, we discuss in detail the expression and function of the different UCP homologs. In addition, the role of UCPs as key regulators of ROS production and redox homeostasis, as well as their influence on the pathophysiology of diabesity and potential role on diabesity-induced male infertility is debated.

## 1. Introduction

**Figure 1:**
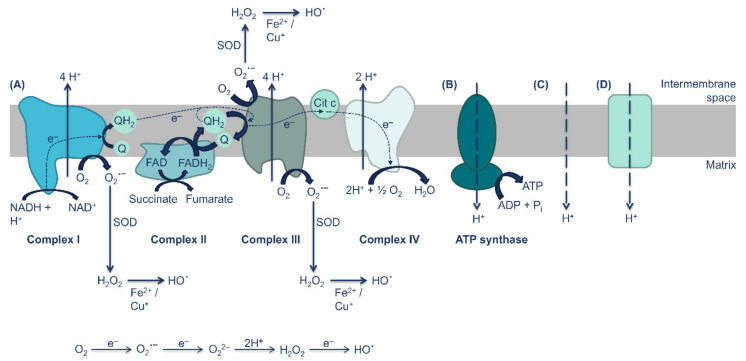
Proton transport and formation of ROS during oxidative phosphorylation. **(A)** In complex I, the oxidation of NADH (generated through glycolysis, β-oxidation, and Krebs cycle) to NAD^+^ transfers two electrons to the complex. Complex I will then transfer the electrons to the quinone reservoir by reducing ubiquinone to ubiquinol. In complex II occurs the oxidation of succinate to fumarate, a reaction that reduces FAD to FADH_2_. FADH_2_ also gives two electrons to ubiquinone, originating FAD and ubiquinol. Ubiquinol is released into the quinone reservoir, joining those from NADH. Ubiquinol transports electrons trough the intermembrane space to complex III, where they are again oxidized to quinones. Complex III transfers electrons to cytochrome c, which moves to complex IV. Complex IV receives the electrons from cytochrome c and transfers them to molecular oxygen, reducing it to water. Same electrons escape to oxygen, which lead to the formation of superoxide anions. Superoxide leads to a cascade of redox reactions, which leads to the formation of other ROS, such as the hydroxyl radical (^•^OH) and hydrogen peroxide (H_2_O_2_). During the transport of electrons through the respiratory chain, protons are pumped from the matrix into the intermembrane space by complexes I, II, and IV. This action creates an electrochemical gradient that is used to **(B)** convert ADP into ATP, by ATP synthase. The proton transport to the mitochondrial matrix can be also made through **(C)** basal proton leak or **(D)** inducible proton leak.

Obesity and diabetes mellitus are perilous health issues that also constitute a huge economic burden worldwide. It is currently estimated that 9.3% of the world population suffers from diabetes mellitus, whereas 39% of adults aged 18 years and over are overweight, and 13% are obese [1,2]. Obesity and diabetes mellitus are intrinsically related to each other, leading to the term “diabesity”. Both conditions are regarded as metabolic diseases due to the impairment of metabolism and augmented levels of reactive oxygen species (ROS), that result in oxidative stress [3]. The biggest source of ROS is the mitochondrial oxidative phosphorylation, which is critical to produce the high quantities of ATP essential for cell survival. However, oxidative phosphorylation is not perfectly coupled to ATP synthesis and some of the energy present in the electrochemical force is dissipated due to the re-entry of protons in the mitochondrial matrix independently of ATP synthase. Indeed, some electrons escape to oxygen (0.2–2%), essentially in complexes I and III, leading to the appearance of reactive oxygen species (ROS) [4,5,6,7]. Although ROS, at physiological levels, are important cell signaling agents, elevated concentrations of these radicals are extremely noxious to cells. Therefore, it is imperative to control their generation [4,5,6,8]. The movement of protons to the mitochondrial matrix without passing through the ATP synthase results in an enormous decrease in ROS production while having a minimal effect on ATP synthesis (a reduced attenuation in the potential difference in the inner membrane of the mitochondria can restrain the formation of H_2_O_2_ by 70%) [7,9,10]. Proton transport to the mitochondrial matrix independent of ATP synthase occurs through two processes: basal proton leak and inducible proton leak (Figure 1). Basal proton leak is not finely regulated and depends only on fatty acid composition of the inner mitochondrial membrane and on the abundance of adenine nucleotide translocase (ANT) [6]. On the other hand, inducible proton leak is finely regulated, with uncoupling proteins (UCPs) playing a crucial role [6].

Herein we discuss in detail the expression and function of the different UCP homologs in human, mouse, and rat. In addition, the role of UCPs as key regulators of ROS production and redox homeostasis and their potential role on the pathophysiology of diabesity and on diabesity-induced male infertility will be discussed.

## 2. Mitochondrial Uncoupling Proteins (UCPs) Expression and Function

**Figure 2:**
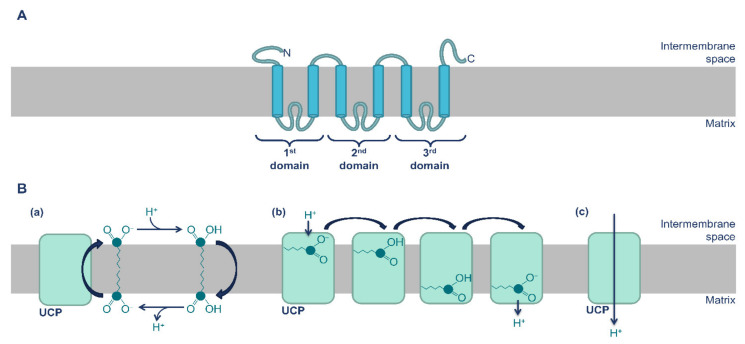
Molecular structure and hypothesized models for proton transport by UCPs. (**A**) UCPs are constituted by three repetition domains, each composed of two α-helix regions. (**B**) The proposed models for proton transport by UCPs are (**a**) flip-flop model; (**b**) cofactor model (transport linked to fatty acids); (**c**) cofactor model (independent transport).

UCPs are integral transmembrane proteins of the inner mitochondrial membrane and members of the mitochondrial anionic transporter family SLC25 [7,9,11,12]. UCPs are constituted by three repetition domains, each composed of two α-helix regions [7,12,13,14,15]. The carboxy and amino-terminal regions are on the intermembrane space while the α-helix regions are linked by long loops that reside on the matrix side of mitochondria (Figure 2A) [7,16,17]. There are two main hypotheses concerning the proton transport mechanism by UCPs: the flip-flop model and the cofactor model (Figure 2B) [18,19]. The flip-flop model occurs in the presence of high concentrations of fatty acids, in which the protonated form of the fatty acid freely crosses the membrane towards the matrix and dissociates the proton in the matrix due to the pH difference (higher in the matrix). The anionic fatty acid returns to the intermembrane space with the help of the UCP, completing the cycle. In the cofactor model, protons can be transported independently or, at a higher rate, linked to the carboxyl group of fatty acids, which operate as prosthetic groups.

UCPs play a central role in regulating the potential of mitochondrial membrane, exhibiting several distinct functions from thermogenesis (dissipating energy in the form of heat) to oxidative phosphorylation or ROS levels regulation [13,18,20]. Currently, six homologs of mammalian UCPs (UCP1-6) have been identified (Table 1), which will be discussed in detail on the following topics.

### 2.1. UCP1

UCP1 is predominantly expressed in brown adipose tissue (BAT), where it constitutes up to 10% of the mitochondrial proteins in this tissue [13,79]. Humans present UCP1 expression almost exclusively in brown adipocytes [21], however, it has also been found to be expressed in white adipose tissue (WAT), on brite or beige adipocytes [22,80] and skin [23]. This expression pattern is shared by mice and rats, where UCP1 expression is almost confined to BAT [24,27], with some studies describing its presence in WAT (including epididymal WAT) [25,28], and mice adrenal gland [26]. Moreover, UCP1 has been identified in smooth muscle cells from mice (including those from the female and male reproductive tracts) [81], and mouse and rat thymocytes [82]. However, these findings have been disputed on later publications [38,83]. Furthermore, the existence of *UCP1* mRNA in human ductus deferens, testis, epididymis, seminal vesicle, and prostate [84] has been predicted.

The main function attributed to UCP1 is the production of heat through thermogenesis [85]. BAT mainly consists of brown adipocytes, cells specialized in thermogenesis that have high expression of thermogenic genes, including UCP1. Exposure to cold leads to adrenergic activation of brown adipocytes and a higher expression of *Ucp1* [86]. As a result, there is an increase in the number and size of mitochondria and the rate of lipolysis is also increased, leading to a higher content of free fatty acids [87]. These free fatty acids activate UCP1, instigating the uncoupling of the respiratory chain and the production of heat [20]. In addition to the cold exposure and adrenergic regulation, including triiodothyronine (T3) and norepinephrine [88], UCP1 activity and expression is also stimulated by superoxide [89], and compounds with a 2-alkenal functional group, including trans-retinoic acid, trans-retinal, trans-2-nonenal, and retinoic acid through a non-adrenergic pathway [90]. In addition, UCP1 also responds to the nutritional status of the organism. Gong et al. reported that *Ucp1* mRNA levels drop with starvation and recover after feeding [91]. UCP1 is also regulated by nucleotides. Interestingly, the binding site of fatty acids to the protein is different from that of nucleotides. Through a FTIR spectra analysis, Chan et al. observed that the addition of nucleotides causes considerable changes in the secondary, and probably tertiary, structure of UCP1 of hamster BAT [92]. In contrast, no secondary or tertiary structure changes were detected upon fatty acid binding. 

### 2.2. UCP2

UCP2, which is 59% identical to UCP1, is probably the most widely expressed UCP homolog [35,93]. Unlike UCP1, UCP2 is presumably ubiquitously expressed throughout the body. In humans, UCP2 expression has been detected in kidneys [29], placenta [31], spermatozoa [32], and brain [35]. In skin [23], lungs [35], WAT, BAT, skeletal muscle [33], and pancreatic β-cells [34], only the mRNA expression has been reported in humans. Widespread UCP2 expression is also observed in rodents, where in mice expression has been identified in thymus [36], kidney [37], mammary gland, stomach [38], macrophages [13], B lymphocytes, T lymphocytes, dendritic cells, neutrophils [45], spleen [39], heart [41], lungs [46], testis [42], and pancreatic α-cells [43], while *Ucp2* mRNA was reported in pancreatic β-cells [34], skin [37], female reproductive tract [38], brain [40], WAT, BAT, skeletal muscle [35], and liver [44]. In rats, UCP2 presence has been described in kidneys [47], neurons [50], microglia [49], heart [51], testis [53], and pancreatic β-cells [54], and its mRNA in liver [48], skeletal muscle [52], WAT, BAT [33], lungs [55], spleen [55], and thymus [56]. Additionally, the existence of *UCP2* mRNA has been predicted in human ductus deferens, testis, epididymis, seminal vesicle, and prostate [84].

The main function attributed to UCP2 comprises the regulation of ROS production and protection against oxidative damage [94,95]. Thus, UCP2 has been studied for its protective effects on several diseases associated with increased oxidative stress, including Alzheimer’s disease, Parkinson’s disease, arteriosclerosis, diabetes mellitus [96,97,98,99]. In addition, UCP2 is reported to have a protective role against stroke and trauma [100], ethanol intoxication [101], brain and cardiac ischemia [102,103], and a regulatory role on inflammation [98,104] and glucose-stimulated insulin secretion [105]. Some studies also report that UCP2 may transport inorganic anions such as aspartate, malate, phosphate, or oxaloacetate [106]. However, it is not known whether the anion transport can be part of the uncoupling cycle or if it is just a side reaction [96]. Interestingly, UCP2 appears to mediate the transport of protons only when specifically activated [96]. UCP2 is activated by high ROS levels, fatty acids, and molecules containing the 2-alkenal functional group [89,96,107]. *Ucp2* mRNA expression is reported to be regulated by glutamine and leptin [108,109]. On the other hand, UCP2 is inhibited by purine nucleotides [89,107].

### 2.3. UCP3

UCP3 shares an 73% identity with UCP2 and 57% with UCP1 [33,60]. In humans, UCP3 is predominantly expressed in skeletal muscle [59] and, to a lesser extent, skin [23], and pancreatic β-cells [58]. An equivalent UCP3 expression pattern is observed in mice which is preferentially expressed in skeletal muscle and BAT [60], and, in a smaller degree, in heart, WAT [61], spleen, and thymus [62]. Likewise, rats have predominant UCP3 expression in skeletal muscle [52], and smaller amounts in heart [64], spleen, and thymus [57]. mRNA has been detected in human heart [33], spleen [57], thymus [57], keratinocytes [23], thyroid, and bone marrow [60], in mice brain, kidney, colon, liver [60], and circulating leucocytes [63], and in rat BAT [52], WAT and kidney [33]. The existence of *UCP3* mRNA was predicted in human ductus deferens, testis, epididymis, seminal vesicle, and prostate [84].

Similar to UCP2, UCP3 also plays a role on the regulation of ROS production and protection against oxidative damage [110,111,112]. In addition, UCP3 is hypothesized to play a protective role against cardiac ischemia [103]. As UCP2, UCP3 is positively regulated by ROS levels [113], fatty acids, molecules containing 2-alkenal functional group [96], and leptin [114], while it is inhibited by purine nucleotides [107]. However, UCP3 also has several resemblances with UCP1. Although it is not normally thermogenic or involved in the adaptive response to cold, UCP3 may be significantly thermogenic under specific conditions, including glucocorticoids or T3 stimulation, and cold exposure [91,96,115]. In addition, *UCP3* mRNA is also regulated by fasting in both human and rat [116,117].

### 2.4. UCP4

UCP4, along with UCP5 and UCP6, are less identical to the other three homologs [15]. UCP4 is predominantly expressed in the central nervous system either from human [7], rat [65] or mouse [66], but its expression has also been detected in other tissues and cells such as Merkel cells, modiolus ear, organ of Corti [67], and chondrocytes [65] of rat, and spinal cord [66], and mast cells [70] of mouse. In human cartilage [65], rat vestibular ganglion [68], heart, lungs, skeletal muscle, kidney, and liver [55] and mouse spiral ganglion, vestibular ganglion, kidney [71], heart, liver, skeletal muscle, WAT [55], and BAT [72], only the mRNA expression was reported. Furthermore, the existence of *UCP4* mRNA in human ductus deferens, testis, epididymis, seminal vesicle, and prostate [84] has been predicted.

The activation of UCP4 attenuates mitochondrial ROS levels [9]. Since the apoptosis of neurons is related to the action of ROS, UCP4 is hypothesized to play neuroprotective role in Alzheimer’s and Parkinson’s disease [118,119]. This uncoupling protein may also be responsible for the observed mitochondrial proton leakage in the brain [120], acting as a thermoregulator in response to cold [69]. Another suggested role for UCP4 in neurons is the regulation of mitochondrial Ca^2+^ signalling [9]. Analogously to the other homologs, UCP4 is inhibited by purine nucleotides and activated by ROS and fatty acids [19]. Calorie restriction and cold induce *Ucp4* mRNA expression in the rat brain as well [20].

### 2.5. UCP5

UCP5 is mainly present in human [73], rat [75] and mouse [74] central nervous system. It is also expressed in other tissues, organs and cells as kidney, heart, lungs, stomach, liver, spleen, skeletal muscle, spinal cord, uterus [73], pituitary, and testis [14] in humans, heart, lungs, adrenals, kidney, gonadal WAT, ovary [14], skeletal muscle, and liver [55] in rat, and spiral ganglion, vestibular ganglion [71], hepatocytes [44], BAT, spleen, intestine, lungs, testis, uterus, and periovarian [14] fat in mouse. Its protein has been detected in mouse brain, heart, kidney, skeletal muscle, WAT, and spinal cord [74]. Additionally, the existence of *UCP5* mRNA has been predicted in human ductus deferens, epididymis, seminal vesicle, and prostate [84].

As UCP4, UCP5 may have a neuroprotective effect against damage related to oxidative stress [74]. This can have implications in several pathological conditions, including Alzheimer’s disease [118], and cortical spreading depression [121]. It is also hypothesized that UCP5 is involved in metabolic rate changes associated with starvation and a high-fat diet [73]. In addition, *Ucp5* mRNA levels decrease during brain hypoxia and increase under hyperoxic conditions [122]. UCP5 is also hypothesized to play a role in thermoregulation induced by cold exposure [73]. Interestingly, UCP5 transports mainly sulphate and thiosulfate [78]. These compounds are products of the degradation of H_2_S, so UCP5 is a potential regulator of the mitochondrial levels of this important signalling molecule. UCP5 also efficiently transports other inorganic anions, including sulphite, phosphate and, to a lesser extent, maleate, oxalate, malonate, malate, citramalate, aspartate, and glutamate [78].

### 2.6. UCP6

*Ucp6* mRNA was found to be mainly expressed in mouse kidneys [30]. It is also considerably expressed in the testis of mice. Its expression has also been detected in other mouse tissues and organs such as WAT, BAT, brain, heart, muscle, liver, lungs, and spleen. The existence of *UCP6* mRNA was predicted in human ductus deferens, testis, epididymis, seminal vesicle, and prostate [84].

UCP6 is upregulated in response to oxidative stress [30]. Although UCP6 seems to have no uncoupling capacity [30], its antioxidant role is hypothesized to be due to the regulation of H_2_S concentration, which at low concentrations inhibits ROS production in mitochondria [123]. In fact, *Ucp6* was found to be upregulated during cancer [124] or the regenerative phase after renal tubular damage [30], which are conditions characterized by high oxidative damage. Moreover, it is assumed that increased *Ucp6* expression is associated with increased mitochondrial activity [30]. Similar to UCP5, UCP6 primarily transports sulphate and thiosulfate, which supports its role as regulator of H_2_S levels [78]. UCP6 is also able to transport the same inorganic anions as UCP5, except for glutamate [78].

## 3. UCPs Are Key Regulators of ROS Production and Redox Homeostasis

ROS are an essential part of the cell function, participating in numerous processes such as immune response, cellular proliferation and differentiation, angiogenesis, aging, and several signaling pathways. They are a natural byproduct of the mitochondria’s oxidative phosphorylation, arising when a single unpaired electron reduces O_2_ at the oxidative chain level, which results in formation of the superoxide anion. However, an unregulated increase in the production of these free oxygen radicals renders the cellular antioxidant system incapable of mitigating their harmful effects, causing a shift in the homeostasis of the redox balance towards oxidative stress, a damaging state that participates on the pathophysiology of several diseases and capable of inducing macromolecular damage towards apoptosis/necrosis [125,126]. It has been proposed that mild uncoupling of the mitochondrial oxidative phosphorylation system might prevent the oversupply of electrons to the various protein complexes of the electron transport chain, diminishing the possibility of electron leaks and interaction with oxygen. Hence, controlled proton conductance via the mitochondrial inner membrane has been suggested as a key regulator of ROS production, through membrane potential modulation [126,127,128,129].

All UCPs homologs are well known for regulating ROS homeostasis, although UCP1 role as an antioxidant remains debatable. Given the extensively described uncoupling activity of UCP1, it would be expected that this protein has an active role in the control of mitochondrial ROS production. However, there are some conflicting data. For instance, Dlasková et al. observed that BAT mitochondria from both wild-type C57BL/6J mice treated with guanosine diphosphate (GDP), an UCP1 inhibitor, and *Ucp1* knockout mice exhibited greater H_2_O_2_ production and higher mitochondrial membrane potential [130]. In accordance with these results, ablation of *Ucp1* in the BAT mitochondria of mice exposed to cold stress leads to higher superoxide production, and subsequently oxidative stress, when compared to wild-type mice [131,132]. Besides, ROS have been shown to be capable of modulating UCP1 activity via sulfonylation of the Cys253 residue, increasing thermogenesis [133]. Conversely, Shabalina et al. reported that UCP1-dependent proton leak is not affected by the oxidative stress byproduct 4-hydroxy-2-nonenal (HNE), and *Ucp1* deletion in mice does not predispose them to higher oxidative damage [134]. In a later study, also using mitochondria from BAT of wild-type and *Ucp1* knockout mice, they determined that UCP1 activity bears no weight in the reduction of ROS emission, except when succinate was endogenously added. Interestingly, the authors also inferred that low membrane potential may not necessarily correlate with lower oxidative stress, challenging the claim that mild uncoupling protects mitochondria against oxidative damage [135].

UCP2 and UCP3 role as modulators of ROS production is extensively described. UCP2 and UCP3 do not display a clear uncoupling activity, so it is unlikely that they regulate superoxide production in a mitochondrial uncoupling-dependent manner. The redox balance mediated by UCP2 was first demonstrated by Nègre-Salvayre et al., where it was shown that mitochondria fractions containing high levels of UCP2 treated with the inhibitor GDP presented a significant increase in H_2_O_2_ generation, whereas the treatment in mitochondria with low expression of UCP2 led to no changes in the oxidative state [56]. Other studies focusing on the overexpression of UCP2, using an adenoviral vector demonstrated that UCP2 plays a protective role against oxidative stress, shielding both cardiomyocytes and endothelial cells from ROS-induced cell death [51,97]. UCP2 neuroprotective role against oxidative stress has also been reported [136,137,138]. Compelling evidence highlights that ablation of *Ucp2* in multiple mice strains induces chronic high levels of ROS in pancreatic cells [139,140,141]. Elevated ROS levels in *Ucp2*-deficient mice also results in higher levels of oxidative stress markers in the liver and delayed hepatic regeneration [95], as well as aggravated atherosclerosis injury [142]. On another note, Arsenijevic et al. observed that *Ucp2* knockout mice infected with *Toxoplasma gondii* presented a stronger infection fighting capacity, when compared to wild type, due to higher ROS emission in macrophages [13]. Due to its ROS suppressing activity, targeted UCP2 inhibition has been proposed as a potential novel treatment for cancer [143,144,145].

Akin to UCP2, it was hypothesized that the overproduction of ROS activates UCP3 through a negative feedback mechanism, diminishing oxidative damage [146]. The presence of superoxide from both endogenous [111,146,147] and exogenous sources [89,148], along with byproducts of lipid peroxidation [149,150] have been shown to induce UCP3-mediated proton leak. In addition, *Ucp3* knockout mice display higher rates of ROS production and are more vulnerable to oxidative damage, including increased vulnerability to ischemia-reperfusion injury [77,110,151,152]. Toime and Brand studied isolated energized skeletal muscle mitochondria from wild-type and *Ucp3* knockout mice and reported that UCP3 decreases the rate of ROS production [151]. Interestingly, these authors treated the isolated mitochondria with FCCP, a well-known chemical uncoupler, which mirrored the UCP3 antioxidant activity. However, inhibition with GDP was ineffective at reducing UCP3-dependent ROS suppression and no differences in the mitochondrial membrane potential were observed, which suggests that UCP3 can modulate ROS production through a membrane potential-independent mechanism. In support to these results, MacLellan et al. demonstrated that moderate overexpression of UCP3 in L6 myocytes does not interfere with basal cellular oxygen consumption or membrane potential despite potentiating its antioxidant activity [153]. UCP3 is also reported to be upregulated in myocytes upon muscle/fiber contraction, suggesting a role on protection against physical exercise-related ROS production [146,154]. Overexpression of UCP3 has also been associated with a protective effect against age-related rise in mitochondrial oxidative damage [155]. On a final note, UCP3-mediated translocation of lipid peroxides to the extramitochondrial medium has also been reported to be a competent strategy in minimizing oxidative stress [156].

Although fewer studies focus on UCP4-6, compelling evidence highlights their role on the regulation of ROS production. Increased levels of *Ucp4* and *Ucp5* mRNA and respective proteins were found to protect neurons against oxidative stress damage [118]. In addition, other studies shown that UCP5 expression decreased the production of superoxide radicals in mouse neurons [74], whereas it protected cells against oxidative stress due to hyperoxic conditions [122]. UCP6 represents an intriguing case. Although UCP6 does not display an uncoupling function, this homolog can be upregulated in response to oxidative stress [30]. It was hypothesized that its antioxidant effect must be due to the regulation of H_2_S levels. Although H_2_S exerts cytotoxic effects when in high concentrations, at low concentrations it exhibits beneficial and protective effects [123]. One of the beneficial effects of low H_2_S concentrations is the inhibition of ROS production in mitochondria through the stimulation of cellular antioxidants [78,123]. Hence, UCP6 seems to contribute to the maintenance of low levels of ROS by keeping the concentration of H_2_S low.

## 4. UCPs Dysregulation Leads to Diabesity

UCPs have a significant influence in mitochondrial bioenergetics, thermogenesis, and control of ROS production, which are all key processes known to progressively become impaired with the onset of metabolic disorders (including obesity and diabetes mellitus). Obesity and diabetes mellitus are closely linked, where a prolonged and persistent positive intake of energy lead to chronic hyperglycemia and subsequent insulin resistance [157]. Even though physical activity accounts for a large percentage of total energy expenditure, resting metabolic rate (RMR) and adaptive thermogenesis are fundamental mechanisms [158,159]. For instance, it is estimated that proton leak through the inner membrane of mitochondria is responsible for 20–30% of the RMR in rats [160]. In fact, alterations in proton conductance and UCPs expression have been implicated in the development of obesity and are considered potential targets for its treatment [161].

UCP1-mediated thermogenesis can increase 12–20% the total daily energy expenditure [162]. However, early studies in *Ucp1* knockout mice were incapable of establish a clear connection between UCP1 deficiency and obesity. Enerbäck et al. were the first to report the absence of an obesogenic effect, as *Ucp1*-deficient mice fed a standard or high-fat diet did not become obese [163]. These findings were later supported by other studies [164,165]. Notably, Feldmann et al. suggested that the housing temperature at which the mice were kept might have confounded some of the results from these studies [166]. Mice housed at a standard temperature (18–22 °C) are under chronic cold stress, which requires an increase in their metabolic rate of 50% to 60% just to sustain body temperature. This increase in energy expenditure might have masked any possible effect of UCP1 in the energy metabolism. In this same study, the authors demonstrated that when *Ucp1* knockout mice were housed at thermoneutral temperature (i.e., 30 °C), they do indeed develop obesity even when fed standard diet. In accordance with these results, more recent studies have also described the obesogenic role of *Ucp1* ablation in mice kept at ~30 °C [167,168], even for notoriously obesity resistant 129 mice strain [169]. Interestingly, it was observed that this strain had markedly higher metabolic rate with increased energy expenditure when compared to other obesity prone strain, the C57BL/6 mice. Raised RMR was explained by higher expression of UCP1 and uncoupling in mitochondria from muscle. After histological analysis it was possible to determine that higher expression of UCP1 correlates with the presence of intramuscular depots of BAT [170]. In addition, Winn et al. reported that *Ucp1* knockout mice fed Western diet and kept at 25 °C developed insulin tolerance, although no significant differences in body weight, visceral adiposity, and energy expenditure in comparison with wild-type mice were observed [171]. Loss of *Ucp1* also causes whitening of BAT [171], decreasing its protective role against the development of insulin resistance, type 2 diabetes, and obesity [172,173]. In fact, an UCP1-dependent role for BAT in glucose homeostasis has been previously described. *Ucp1*-deficient mice lack BAT glucose uptake after norepinephrine administration, suggesting that sympathetic stimulation leads to UCP1 mediated glucose disposal by BAT [174]. Notably, BAT transplant to *Ucp1* knockout mice was able to eliminate glucose intolerance [175]. Furthermore, stimulation of UCP1 dependent BAT thermogenesis, either through cold or diet, has been shown to increase energy expenditure via catabolic acceleration. When active, BAT oxidizes significant amounts of metabolic substrates such as stored fatty acids, as well as plasma glucose and triglycerides. Thus, through reduction of the circulating glucose and lipids in order to supply the energy costly thermogenesis, BAT is a tissue of great interest to treat metabolic disorders such as obesity and type 2 diabetes mellitus [87,167,168,171,176]. In accordance with these results, Vázquez et al. reported that Zücker diabetic fatty rats, an experimental model for obesity and type 2 diabetes mellitus, exhibited significantly lower levels of UCP1 protein and mRNA expression in BAT when compared to their lean littermates. Interestingly, treatment with melatonin, which has been described has having an anti-obesity effect, was able to restore not only UCP1 expression but also its functionality in obese rats. This increase in UCP1 expression and function resulted in higher thermogenic activity, which was linked with enhanced energy expenditure and overall improved metabolic state of treated rats [177]. Further evidence for the UCP1 antidiabetic and anti-obesity effect comes from studies in transgenic mice models overexpressing UCP1. Transgenic mice with upregulation of UCP1 in WAT showed resistance to diet-induced obesity even when fed a high-fat diet [178,179]. Moreover, several studies have linked ectopic overexpression of UCP1 in the skeletal muscle with higher energy expenditure, lean phenotype, lower levels of glucose, insulin, cholesterol, and adiposity, enhanced lipid metabolism and glucose transport, and improved responsiveness to insulin [180,181,182,183,184].

UCP2 has also been implicated in the pathogenesis of diabesity, almost since its discovery. A study by Zhang et al. observed that *Ucp2* knockout mice had improved glucose-stimulated insulin secretion (GSIS), indicating that UCP2 activity negatively regulates insulin secretion [105]. The authors also reported that UCP2 was significantly upregulated in isolated pancreatic islets of a model of obesity-induced diabetic mice (ob/ob mice), when compared to control. Interestingly, ob/ob mice lacking *Ucp2* had enhanced β-cell responsiveness to glucose, decreased levels of glycemia and restored first phase insulin secretion. Overall, these results seem to indicate that obesity-dependent induction of UCP2 in pancreatic β-cells has a pathogenic effect towards the development of insulin resistance and in turn diabetes mellitus. In accordance with this hypothesis, overexpression of UCP2 in isolated β-cells was reported to impair GSIS [34,54,185], while inhibition with genipin, an UCP2 inhibitor, improved glucose secretion [186]. Desouza et al. observed that partial inhibition of UCP2 with an antisense oligonucleotide in two mice models of type 2 diabetes mellitus, ob/ob mice and diet-induced obese and diabetic mice, led not only to improvements in insulin secretion in β-cell but also enhanced insulin action in peripheral tissues (eg. adipose tissue) [187]. Saleh et al. shown that acute knockdown of UCP2 in isolated pancreatic islets from lean mice increased in vitro insulin secretion but failed to improve GSIS in isolated pancreatic islets from ob/ob mice [188]. Nevertheless, dissenting results have highlighted the possibility of the confounding effects of genetic background and systemic absence of protein activity in studies with *Ucp2*-ablated mice (detailedly reviewed in [189]). In addition, early reports negatively correlated UCP2 induction with β-cell ATP levels [34,54,105,186,188]. A study by Affourtit and Brand using acute knockdown of *Ucp2* in clonal β-cells, INS-1E cells, reported that UCP2 is responsible for the high mitochondrial proton conductance observed in this model [190]. ATP is a powerful stimulus for insulin secretion, which led the authors to speculate that UCP2 modulates GSIS in pancreatic islets through the coupling efficiency of oxidative phosphorylation. In fact, this hypothesis is shared by several other authors [54,105,187]. Growing evidence also has revealed the pivotal role of ROS production in β-cell function. Glucose-dependent ROS production and overall changes in the redox status of pancreatic islets have been shown to stimulate insulin production and secretion, which in turn can be suppressed through antioxidative activity. Therefore, a role for ROS in controlling β-cell function under pathological conditions, such as obesity and diabetes mellitus, has been proposed [191,192,193]. In support, several studies focused on the potential role of UCP2 in GSIS through the control of mitochondrial ROS production and protection against oxidative stress. For instance, *Ucp2* ablation in INS-1E insulinoma cells resulted in improved GSIS, which can be mimicked using the cell-permeative antioxidant MnTMPyP [194]. A study using a refined model of β-cell specific *Ucp2* knockout reported that *Ucp2*-ablated pancreatic islets had increased ROS levels correlating with increased GSIS [140]. Interestingly, while no changes in the uncoupling respiration rates and ATP/ADP ratio were observed, *Ucp2*-deficient islets exhibit mildly increased mitochondrial membrane potential [140]. Taken together, these findings suggest that *Ucp2* knockout increases intracellular ROS and thus stimulate insulin secretion. Thus, UCP2 might be seen as a double-edged sword: decreasing its activity seems to improve GSIS, however it leaves insulin-secretion β-cell susceptible to oxidative damage, which suggests a finely regulated activity.

UCP3 plays an active role in ROS production and fatty acid oxidation, which makes it a candidate for better understanding the pathophysiology of metabolic disorders such as obesity and type 2 diabetes mellitus. Experimental evidence from multiple studies have proposed UCP3 as a modulator of energy metabolism and a possible protective role in obesity. For instance, transgenic mice overexpressing UCP3 fed a fatty diet exhibit lower body weight, decreased adipose tissue, and triglyceride accumulation [195,196,197]. Furthermore, moderate upregulation of UCP3 can mimic the beneficial consequences of physical exercise, promoting fatty acid oxidation, increasing energy expenditure and consequentially increase weight loss [198]. In contrast, studies using *Ucp3*^−/−^ mice failed to demonstrate an obesogenic effect for *Ucp3* ablation [77,199]. More surprisingly, a recent study by Lomax et al. linked *Ucp3* deficiency with protection from diet-induced obesity [200]. However, it is important to highlight that housing of animals in conditions of chronic thermal stress may have conflicting results. In regard to diabetes mellitus, overexpression of UCP3 improves glucose tolerance and protected the animals against fat-induced insulin resistance [201,202]. Studies focusing in *Ucp3* ablation have conflicting evidence as *Ucp3*^−/−^ animals fed a fatty diet exhibited either enhanced [200], decrease [203] or unaltered [77] insulin resistance. In humans, high glucose exposure decreased UCP3 protein expression in isolated islets, whereas overexpression of UCP3, contrary to UCP2, improves GSIS [58]. Concurrently, clinical studies have reported lower expression of *UCP3* mRNA and protein in prediabetic and diabetic individuals, when compared to healthy individuals [204,205,206]. Interestingly, both 8-week treatment with rosiglitazone and physical exercise training were able to restore UCP3 levels in diabetic patients [205].

## 5. The Potential Role of UCPs on Diabesity-Induced Male Infertility

The link between oxidative stress and male factor infertility is well documented since it was first described by MacLeod in 1943, where a connection between H_2_O_2_ production and loss of spermatozoa mobility was observed [207]. In fact, clinical studies have reported that 30% to 80% of infertile men exhibit supraphysiological levels of seminal ROS [208,209,210,211,212,213]. Furthermore, a study comparing ROS levels in semen from proven donor (those who resulted in a pregnancy) and infertile men, using chemiluminescence assay, reported that fertile men possessed reduced ROS levels [214]. Several studies have already established a correlation between oxidative stress and poor sperm parameters, including reduced sperm concentrations and motility, aberrant morphology, as well as increased DNA fragmentation [215,216,217,218,219].

Interestingly, ROS have been described as a double-edged sword for spermatozoa. ROS have a crucial influence in sperm physiology and spermatogenesis, as well as in the fertilization process. Seminal ROS participate as a secondary messenger in several pathways necessary for spermatozoa hyperactivation, capacitation, sperm–oocyte fusion, chemotaxis, acrosome reaction, and sperm head decondensation [220,221,222,223]. In addition to endogenous sources, ROS may also arise from exposure to pollutants, radiation, and drugs [224]. An imbalance in the redox homeostasis, as a result of increased ROS production or a deficient antioxidant system (or both), result in pathological oxidative damage to spermatozoa. Interestingly, it has been recently shown that antioxidant mechanisms, as is the case of Sperm Nuclear Basic Proteins (SNBP), when in contact with certain pollutants such as heavy metals can overturn their protective role and contribute to oxidative damage, which highlights how vulnerable spermatozoa are to oxidative stress [225,226]. Even though infertile men present lower seminal plasma antioxidant capacity [79,227], oxidative damage in spermatozoa is mostly associated with exacerbated levels of ROS as opposed to impaired superoxide scavenging activity [211]. Loss of motility is one of the major consequences of oxidative stress in spermatozoa [228], arising from a combination of reduced membrane fluidity, due to dysregulated lipid peroxidation [229], and defective axonemal phosphorylation [230,231,232]. Furthermore, oxidative stress has been associated with spermatozoa DNA damage. Disruption in sperm genomic integrity clinically relates to augmented teratozoospermia, impaired oocyte fertilization, abnormal embryonic development, and increased miscarriage rates [233,234,235].

As previously mentioned, individuals who suffer from obesity or diabetes mellitus present augmented levels of ROS, which results in oxidative stress and ultimately subfertility or even infertility. In fact, the deleterious effects of diabetes mellitus and obesity in male reproductive function are well established. Patients suffering from diabetes and/or obesity exhibit poor sperm parameter, decreased motility, abnormal morphology, reduced density, and higher damage to both nuclear and mitochondrial sperm DNA [236,237]. It was also reported that obese men have a higher mean scrotal temperature than healthy individuals, which can be related to male infertility [238,239]. Besides, hormonal dysfunctions that decrease steroidogenesis in Leydig cells, resulting in hypogonadism, and alterations in the body energy metabolism disturb the normal function of testis [157,237]. On the other hand, weight loss and improved lifestyle has been associated with better sperm parameters [240], although a series of studies by Crisóstomo et al. demonstrated that the consumption of a high fat diet during childhood permanently alters testicular metabolism which led to an irreversible decline in sperm quality [241,242]. Glucose metabolism is of great importance in the testis as sperm cells are highly sensitive to glucose concentrations. Accordingly, glucose transport through the blood-germ barrier is a highly controlled process. Hyperglycemia is particularly harmful to spermatogenesis being correlated with poor reproductive outcomes [243]. Sertoli and Leydig cells as well as spermatozoa have been shown to be sensitive to insulin, therefore, glucose homeostasis is necessary for male reproductive function [244]. Furthermore, both obese and diabetic patients have been reported to exhibit elevated ROS in testis and seminal fluid, which is, as described above, extremely detrimental [245,246].

Further understanding of the nefarious impact of ROS production and antioxidant mechanism in male related infertility will surely lead to more focused treatment and accurate diagnostic, as well as better outcomes for subfertile and infertile men. Hence, UCPs by virtue of their antioxidant activity may represent a clinically relevant target to improve reproductive outcomes. UCPs seem to play an important part in mediating the deleterious effects of diabesity in male infertility and subfertility, however little is known about their function in the male reproductive tract. Given the extensively described role of UCPs as key regulators of the mitochondria redox state, it should not come as a surprise that most of the experimental data comes from studies on the activity of UCPs in the testis/sperm ROS production, more precisely UCP2. Zhang et al. reported that UCP2 protects germ cells from hyperthermia-derived ROS production. Furthermore, *Ucp2* mRNA expression was increased in the mouse testis of experimental cryptorchidism. Hyperthermia in the testis arises from multiple pathological conditions such as varicocele, cryptorchidism, or obesity, and correlates with exacerbated spermatozoa apoptosis [42]. In another study, it was found that ROS production is the main mechanism behind hyperthermia-induced sperm cell apoptosis. Acute exposure of mouse testicles to heat (43 °C for 5 min) was sufficient to induce a 6-fold increase in UCP2 expression. Furthermore, overexpression of UCP2 in mouse GC-2 cell line, a model for germ cell differentiating, improved the survival rate after exposure to the ROS-inducing agent menadione [42]. Moreover, older mice exhibit increased UCP2 activity and proton leak in the testis. Testicular age involves higher rates of mitochondrial dysfunction which correlates with increased oxidative stress, therefore, UCP2 upregulation in older testis might be a protective mechanism [53]. In humans, UCP2 expression was found to be significantly increased in spermatozoa from donors with normal sperm motility when compared to asthenospermic men [32]. The link between UCP2 and ROS production was further highlighted through treatment of spermatozoa with genipin, which resulted in elevated levels of mROS and decreased sperm motility. It should be noted that it was recently reported by Kreiter et al. that genipin is incapable of specifically inhibit UCP2, but rather is a nonspecific inhibitor of UCPs activity [247]. Additionally, treatment of spermatozoa with H_2_O_2_ was shown to induce UCP2 expression [32]. Although the experimental data on the role of UCPs in the male reproductive function is scarce, given their presence in testis and their established function in other tissues it should be expected that they are important modulators of testicular bioenergetics and redox state. Therefore, they are enticing targets for further research in the crosstalk between diabetes mellitus and obesity and their secondary effects in male reproduction.

## 6. Conclusions and Future Perspectives

UCPs are widely expressed throughout the organism and play an important role on ROS production and homeostasis. UCP1 is responsible for thermogenesis, but the other homologs display broader functions, including insulin release and regulation of oxidative phosphorylation. Due to their functions, UCPs take part in the pathophysiology of diabesity, where its altered expression and/or dysfunction is associated with the onset of the disease. Studying UCPs expression and function will lead to a better understanding of the pathophysiology of metabolic diseases and potentially to novel therapeutic targets. As mentioned above, diabesity is positively associated with high oxidative stress and, consequently, to male subfertility or even infertility, which is one of the most overlooked comorbidities. Working towards a better understanding of the relationship of UCPs activity with male reproductive function could lead to the development of novel fertility therapies and better reproductive outcomes. However, not much is known concerning the expression and function of UCPs in the male reproductive tract. Further studies are needed to disclosure the role of UCPs in male fertility and redox signaling and homeostasis during spermatogenesis and spermatozoa physiology.

## Figures and Tables

**Table 1:** Expression and hypothesized functions of all mitochondrial uncoupling proteins (UCPs) homologs.

Isoform	Localization	Putative Function	References
**UCP1**	**Human** brown adipose tissue, white adipose tissue, keratinocytes, sweat glands, sebum glands, hair follicles, and granular layer of the epidermis	Non-shivering thermogenesis, metabolic and bioenergetic regulation, modulation of ROS production	[21,22,23]
	**Mouse** brown adipose tissue, white adipose tissue, and adrenal gland		[24,25,26]
	**Rat** brown adipose tissue and white adipose tissue		[27,28]
**UCP2**	**Human** proximal tubular cells, cytotrophoblasts, syncytiotrophoblast, lungs *, keratinocytes *, skin fibroblasts *, spermatozoa, white adipose tissue *, skeletal muscle *, brown adipose tissue *, and pancreatic β-cells *, brain	Modulation of ROS production, insulin sensitivity and secretion, lipid and glucose metabolism, control of mitochondrial Ca^2+^-uptake, inflammation, immunomodulation	[23,29,30,31,32,33,34,35]
	**Mouse** thymocytes, tubular epithelial cells, granulosa cells *, theca cells *, endometrium glandular epithelium cells *, uterine glands *, oviduct mucosa epithelial cells *, mammary gland, stomach, macrophages, splenocytes, B lymphocytes, T lymphocytes, dendritic cells, neutrophils, microglial cells *, neurons *, cardiomyocytes, lungs, testicular germ cells, testicular interstitial cells, white adipose tissue *, skeletal muscle *, brown adipose tissue *, pancreatic β-cells *, pancreatic α-cells, and hepatocytes *		[13,34,35,36,37,38,39,40,41,42,43,44,45,46]
	**Rat** proximal tubular cells, Kupffer cells *, microglial cells, neurons, cardiomyocytes, skeletal muscle *, testis, brown adipose tissue *, white adipose tissue *, pancreatic β-cells, lungs *, spleen *, and thymus *		[33,47,48,49,50,51,52,53,54,55,56]
**UCP3**	**Human** skeletal muscle, heart *, spleen *, thymus *, keratinocytes *, skin fibroblasts, sweat glands, hair follicles, stratum basale of the epidermis, pancreatic β-cells, thyroid *, and bone marrow *	Modulation of ROS production, insulin secretion, lipid metabolism, protection against lipotoxicity, control of mitochondrial Ca^2+^-uptake, immunomodulation	[23,33,57,58,59,60]
	**Mouse** skeletal muscle, brown adipose tissue, brain *, kidney *, colon *, liver *, heart, white adipose tissue, thymocytes, splenic lymphocytes, and peripheral naive CD4+ T cells *		[60,61,62,63]
	**Rat** skeletal muscle, brown adipose tissue *, white adipose tissue *, kidney *, spleen reticulocytes, spleen monocytes, spleen lymphocytes, thymocytes, and heart		[33,52,57,64]
**UCP4**	**Human** Purkinje cells, most of the brain tissues *, and cartilage *	Regulation of oxidative stress, thermoregulation, protection against mitochondrial Ca^2+^ overload	[7,9,15,65]
	**Rat** Merkel cells, modiolus ear (fibrocyte, satellite cells), organ of Corti (spiral ganglion neurons, supporting cells, hair cells), brain (pyramidal cells of hippocampus and cortex, Purkinje cells of cerebellum, neurons of hippocampus, substantia nigra, striatum, neocortex *), chondrocytes, vestibular ganglion *, heart *, lungs *, skeletal muscle *, kidney *, and liver *		[7,55,65,66,67,68,69]
	**Mouse** brain (neurons, astrocytes, cortex, brainstem, cerebellum), spinal cord, mast cells, spiral ganglion *, vestibular ganglion *, kidney *, heart *, white adipose tissue *, brown adipose tissue *, skeletal muscle *, and liver *		[55,66,70,71,72]
**UCP5**	**Human** kidney *, heart *, lungs *, stomach *, liver *, spleen *, skeletal muscle *, brain (cerebellum, cortex, medulla, occipital pole, frontal lobe, putamen, amygdala, caudate nucleus, hippocampus, substantia nigra, thalamus, corpus callosum) *, spinal cord *, pituitary *, uterus *, and testis *	Regulation of oxidative stress, thermoregulation, transport of metabolites, regulation of mitochondrial metabolism	[14,73,74]
	**Rat** heart *, lungs *, adrenals *, kidney *, gonadal fat *, ovary *, brain (striatum, cortex, hippocampus, cerebellum) *, skeletal muscle *, and liver *		[14,55,75]
	**Mouse** heart, kidney, skeletal muscle, white adipose tissue, spinal cord, brain (neurons, astrocytes, cortex, thalamus, hippocampus, substantia nigra, cerebellum, basal ganglia, hypothalamus *, amygdala *, neocortex *, caudate putamen *), spiral ganglion *, vestibular ganglion *, hepatocytes*, brown adipose tissue *, spleen *, intestine *, lungs *, testis *, uterus *, and periovarian fat *		[14,30,44,71,72,74,76,77,78]
**UCP6**	**Mouse** kidney (proximal tubules, distal tubules, surrounding nephron segments, glomeruli, medullary part of the loop of Henle, collecting duct) *, white adipose tissue *, brown adipose tissue *, brain *, heart *, muscle *, liver *, lungs *, spleen *, and testis *	Regulation of oxidative stress, transport of metabolites, regulation of mitochondrial metabolism	[30,74]

* Only mRNA has been reported or predicted based on mRNA microarrays.
